# Malignant Melanoma: Landscape of Molecular Markers

**DOI:** 10.3390/biomedicines14010157

**Published:** 2026-01-12

**Authors:** Melanie Winter, Silvana Ebner, Viola Baum, Kati Kiil, Marc-Alexander Rauschendorf, Peter J. Wild

**Affiliations:** 1 Dr. Senckenberg Institutes of Pathology and Human Genetics, University Hospital, Goethe University Frankfurt, 60590 Frankfurt, Germany; 2 University Hospital Frankfurt MVZ GmbH, 60590 Frankfurt, Germany; 3 Molecular Health, Kurfuersten-Anlange 21, 69115 Heidelberg, Germany; marc.rauschendorf@molecularhealth.com

**Keywords:** melanoma, *BRAF*, *NRAS*, personalized therapy, next-generation sequencing, NGS

## Abstract

**Background:** In melanoma diagnostics key molecular markers, such as *BRAF*, *NRAS*, and *KIT* mutations also paved the way for targeted therapies. Immunotherapies, including immune checkpoint inhibitors like anti-CTLA-4 and anti-PD-1/PD-L1, have revolutionized treatment, improving survival outcomes for advanced-stage melanoma patients. Despite these advances, challenges such as resistance to targeted therapies and variability in patient responses to immunotherapy remain critical issues. The purpose of the project is to characterize the molecular landscape of a set of 28 malignant melanomas using next-generation sequencing, identify the prevalence and nature of class 3–5 variants (e.g., *NRAS*, *BRAF*, *KIT*, *TP53*), assess the genetic complexity and molecular patterns, and use these insights to inform personalized therapies and optimize patient stratification for potential combination strategies (targeted therapy followed by immunotherapy). **Methods:** We analyzed a set of malignant melanoma of the skin of 17 women (61%) and 11 men (39%) at the age of 23 to 85 years (median: 63 years) by tumor-only next generation sequencing. **Results:** 22/28 cases (79%) present a pathogenic or likely pathogenic variant with an allelic frequency of ≥5%. In total 42 distinct somatic pathogenic or likely pathogenic variants with an allelic frequency of ≥5% could be detected. The most frequent pathogenic molecular alteration in these melanomas were found in *NRAS* (25%) and *BRAF* (25%). The most frequent molecular alteration of unknown significance was found in *FANDC2* (46%), *NOTCH3* (39%), *ARID1A* (32%), *PMS2* (32%), *POLE* (29%), *NOTCH1* (29%), *TSC2* (25%), *SMARCA4* (25%), *ATR* (25%) and *TERT* (21%). **Conclusions:** While *NRAS* and *BRAF* were the most frequent actionable alterations (each 25%), a broad spectrum of variants of unknown significance (e.g., *FANDC2*, *NOTCH3*, *ARID1A*, *PMS2*, *POLE*, *NOTCH1*, *TSC2*, *SMARCA4*, *ATR* and *TERT*) also predominates, underscoring the genetic complexity of melanoma. These variants complicate clinical decision-making because their contribution to tumorigenesis, therapeutic response, and prognosis remains uncertain. Nevertheless, these variants also offer a valuable resource for future research, as they may uncover novel pathogenic mechanisms or therapeutic targets once their significance is elucidated. Integrating comprehensive genetic profiling with immunologic markers can enhance patient stratification and support rational, potentially synergistic strategies, such as combining targeted therapies with immunotherapy, to optimize clinical outcomes. This study is limited due to a small cohort and limited available clinical data. Larger cohort studies and prospective clinical trials are necessary to validate and explore the interplay between molecular and immune biomarkers as well as general biological mechanism in paving therapeutic way in melanoma.

## 1. Introduction

Melanoma, an aggressive skin cancer originating from melanocytes, represents a significant global health concern due to its high metastatic potential and increasing incidence [1]. This malignant tumor of melanocytes can be challenging to diagnose—especially in atypical or amelanotic cases [2]. Immunohistochemistry (IHC) plays a critical role in confirming diagnosis and distinguishing melanoma from other neoplasms, such as carcinomas, lymphomas, or sarcomas. Currently, S100, SOX10, HMB-45, and Melan-A are used in routine diagnostics [3,4].

Early detection and prompt treatment are critical for favorable outcomes; however, the prognosis for advanced-stage melanoma remains poor. Standard treatment includes the complete excision of the primary lesion and margins. In patients with an increased metastatic risk, the sentinel lymph node is also removed. The Breslow invasion depth, ulceration of the primary lesion, and involved sentinel lymph nodes determine the prognostic value. Systemic therapy plays an important role in the adjuvant setting and for inoperable tumors. Traditional diagnostic and therapeutic approaches have demonstrated limited efficacy, necessitating the development of more precise strategies. Personalized therapy for malignant melanoma represents a paradigm shift in the management of this aggressive cancer [5,6]. 

In recent years, advances in molecular biology have transformed our understanding of melanoma pathogenesis. The identification of key molecular markers, such as mutations in *BRAF*, *NRAS*, and *KIT*, has not only deepened insights into tumor biology but has also established a foundation for targeted therapies [7,8]. Beyond the mentioned melanoma drivers understanding melanoma etiology has advanced with recognition of skin photodamage and UV-induced carcinogenesis as key determinants of tumor development. Recent review synthesizes how environmental factors—UV exposure, skin phototype, and cumulative photoaging—shape mutational signatures and tumor biology, and how these signals intersect with genomic alterations. For instance, UV-associated mutational patterns and copy-number changes can influence downstream pathways such as MAPK and PI3K–AKT, providing a contextual framework for interpreting somatic and germline variants. Integrating environmental risk with molecular data may refine the prioritization of variants of unknown significance (VUS) and enhance translational relevance [9].

Moreover, epigenetic modifications, gene expression profiles, and proteomic analyses have provided additional layers of complexity and opportunity in melanoma research. These molecular insights have led to the development of innovative diagnostic tools and more effective treatment modalities as summarized in Table 1.

The integration of molecular markers into therapeutic decision-making has revolutionized the management of melanoma. Targeted therapies, such as BRAF and MEK inhibitors, offer personalized treatment options for patients with specific genetic alterations, while immunotherapy, including checkpoint inhibitors like anti-PD-1 and anti-CTLA-4, has significantly improved survival rates for advanced melanoma [6,10,11,12]. Despite these advancements, challenges persist, including therapeutic resistance, variability in patient responses, and the need for reliable prognostic and predictive biomarkers.

According to the recommendation of distinct guidelines (ESMO [13], NCCN [14], S3 [15] and Oncopedia [16]) testing for *BRAF* (strongly recommended by ESMO, NCCN, S3 and Oncopedia), *NRAS* (strongly recommended by NCCN and S3), and *KIT* (strongly recommended by NCCN, S3 and Onkopedia) mutations is essential to inform treatment decisions in resectable or unresectable stage II and IV melanoma. *BRAF* mutation status is the most critical marker for targeted therapies. *NTRK* fusions are very rare (<1%) in melanoma but are important because targeted therapies are available [17]. Testing is recommended by ESMO, NCCN, S3 and Oncopedia in selected cases, especially in advanced disease. PD-L1 expression is routinely tested to assist in immunotherapy planning, although it is not the sole determinant for treatment.

This paper aims to explore the current role of molecular markers in the diagnosis and treatment of melanoma. We discuss the current state of molecular characterization in routine diagnostics, its impact on therapeutic strategies, and emerging trends in biomarker discovery. Integrating molecular insights with clinical practice pave the way to more effective, individualized approaches to combat melanoma and improve patient outcome. Therefore, small cohorts also play an important role, as they are ideal for the discovery of rare VUS or local population differences.

## 2. Materials and Methods

### 2.1. Collective of Patients/Inclusion and Exclusion Criteria

28 patients with a clinically malignant melanoma of the skin were included. To detect somatic mutations formalin-fixed paraffin-embedded (FFPE) tumor samples, collected from 2017 to 2022, were macrodissected.

Inclusion criteria:

➢ Minimum tumor content of 15%;
➢ Histology: malignant melanoma of the skin.

Exclusion criteria:

➢ Samples with tumor content below the threshold were excluded (*n* = 0);
➢ Insufficient sequencing parameters as described below (*n* = 5);
➢ Uveal melanoma were excluded (*n* = 4).

### 2.2. Pre-Analytical Quality Control of Tissue Samples

Tissue samples were collected from surgical resections or biopsies under standardized conditions. Thus, the time interval between excision and fixation (cold ischemia time) was maintained at ≤30 min, and samples were kept on ice or at 4 °C during transport to minimize degradation. Each specimen was labeled with a unique identifier to ensure full traceability throughout processing. Fixation was performed using 10% neutral buffered formalin (equivalent to 4% formaldehyde) at room temperature (18–25 °C). The fixative-to-tissue volume ratio was at least 10:1 to ensure adequate penetration, and fixation was carried out for 12–24 h (acceptable range: 6–48 h). Over-fixation and under-fixation were avoided to preserve nucleic acid integrity and antigenicity. After fixation, tissues were processed through graded ethanol (70%, 80%, 95%, and 100%) for dehydration, followed by clearing in xylene or an appropriate substitute, and infiltration with paraffin wax (melting point 56–58 °C). Processing temperatures were kept below 60 °C to prevent heat-induced nucleic acid degradation. Equipment used for tissue processing and embedding was routinely maintained and calibrated at least once per year. Formalin-fixed, paraffin-embedded (FFPE) blocks were stored at room temperature (18–25 °C) in a dry, dark environment. 3–8 FFPE slides (3 µm thick), depending of the sample size, were used for hematoxylin and eosin (HE) staining and further molecular analysis. For quality assurance, a post-H&E and an H&E staining of the scraped section are prepared from each FFPE block used for molecular analyses to verify the identity and representativeness of the analyzed tissue. A consultant pathologist did this quality control.

NGS Diagnostics: The QIAamp^®^ DNA Micro Kit (50) (Qiagen N.V., Venlo, The Netherlands) were used for DNA extraction according to the manufacturer’s instructions. The concentration was measured using the Qubit™ 4.0 system and dsDNA HS Assay Kit according to the protocol [18]. DNA samples with a concentration of ≥1 ng/uL were included. The Oncomine ThermoFisher NGS Workflow was applied for the variant detection. SeraSeq^®^ Tumor Mutation DNA Mix v2 AF10 (SeraCare, Milford, MA, USA) [19] was used as a control for quality assurance. The Ion Chef^TM^ system (ThermoFisher Scientific, Waltham, MA, USA) [20] was used for clonal amplification and chip loading. The subsequent sequencing was performed on the Ion GeneStudio^TM^ S5 (ThermoFisher Scientific, Waltham, MA, USA) [21]. The Oncomine Comprehensive DNA Assay v3 (ThermoFisher Scientific, Waltham, MA, USA) was applied according to the manufacturer’s protocol. For sequencing analysis following criteria had to be met:

- % Q30 bases: >95%;
- Total number of reads: >50,000,000;
- Aligned reads: >95%;
- Coverage 500x: >95%.

As recommended by the ACMG/AMP variants with an allele frequency of ≥5% were reported and classified according to the evidence codes for benign and pathogenic criteria [22].

As visible in Table 2 distinct single-nucleotide variants (SNVs) and copy number variations (CNVs) can be detected.

For variant detection, the data were analyzed with the Ion Reporter™ software (version 5.12.0.0); filter chains Oncomine Variants 5.12 and Oncomine Extended 5.12 were used. Class 3–5 variants (class 3: variants of unknown significance; class 4: likely pathogenic; class 5: pathogenic) were identified by the alignment on the reference genome hg19 (GRCh37) available at https://www.ncbi.nlm.nih.gov/ (accessed on 20 December 2024). Duplicate reads were automatically identified and removed during alignment to reduce PCR artifacts. To mitigate the known Ion Torrent limitation of indel errors in homopolymer regions, all variants in such regions were manually reviewed using the Integrative Genomics Viewer (IGV), and only high-confidence variants were retained. Variant calling was performed in Ion Reporter Software (Thermo Fisher Scientific, Waltham, MA, USA) using the default somatic workflow, which requires a minimum of 20 variant-supporting reads and a variant allele frequency (VAF) threshold of ≥5% and a coverage of at least 500x for variant inclusion. Cases were analyzed using MH Guide (v6.3, Molecular Health, Germany), a CE-marked (IVDR 2017/746) tertiary NGS analysis software. These cutoffs were selected to balance sensitivity and specificity, minimizing false positives while reliably detecting clinically relevant mutations in heterogeneous tumor samples. The thresholds are consistent with Ion Torrent and Oncomine recommendations, and align with best practices in NGS diagnostics, including guidelines from the ACMG (American College of Medical Genetics and Genomics), AMP (Association for Molecular Pathology), CAP (College of American Pathologists), and ESMO (European Society for Medical Oncology), which recommend sufficiently deep coverage and AF thresholds to ensure accurate somatic variant calling in oncology.

MH Guide identifies reportable variants and provides clinical interpretation, including potentially effective, ineffective, or high-risk medications. It offers variant annotation, classification, and interpretation based on a curated, peer-reviewed evidence database [23]. The variant classification was manually reviewed according to the online databases ClinVar [24] and Cosmic [25]. Other databases used for variant interpretation were gnomAD v2.1.1 [26], OncoKB [27], dbSNP [28], and cBioPortal v6.4.1 [29] (available online). To achieve a consistent approach of naming all variants, sequence variant nomenclature was carried out in concordance with the guidelines by the Human Genome Variation Society (HGVS) [30].

### 2.3. CVI (Curated Variant Information) Scores for Predictive Biomarkers

The IVDR-certified tertiary analysis software MH Guide (Molecular Health, Heidelberg, Germany) was used to annotate and classify patient-specific genomic alterations and to match them with potential treatment options based on published evidence. MH Guide provides multiple established clinical tiering systems to ensure a quality-assured interpretation of the clinical actionability of molecular targets. These systems differ in their clinical objectives, evidence inclusion, and scope of application. Available frameworks include the AMP/ASCO/CAP tiering system, ESMO ESCAT evidence levels, the German NCT evidence grading [31,32,33] and the proprietary MH Curated Variant Information (CVI) score.

For the present analysis, treatment options were considered if they had FDA or EMA approval for the patient’s condition (MH CVI = 7), or if there was at least one large-scale study or approval for another cancer type (MH CVI = 6). A score of 6, for example, can be attributed based on evidence from a patient-specific indication or from another indication, with specifically matched CVI content for each. An MH CVI score of 7 corresponds to AMP/ASCO/CAP Tier IA, while an MH CVI score of 6 corresponds primarily to AMP/ASCO/CAP Tiers IB and IIC. MH CVI scores range from 1 (preclinical evidence) to 7 (clinically approved therapy). MH CVI scores, together with MH CVI narratives, offer a comprehensive combination of indications, biomarkers, variant-drug relations, and drug approvals.

All MH CVI data are curated and reviewed by an internal team of PhD-level experts at Molecular Health under IVDR-compliant and ISO 13485 [34] conforming SOPs. Each CVI entry undergoes multiple rounds of scientific and medical review before approval.

The proprietary MH CVI scoring system describes biomarker evidence levels as follows:

CVI score 7 (Clinically approved): The biomarker has been approved by a regulatory agency such as the FDA, EMA and/or other relevant authorities to predict a specific effect of the drug (i.e., to be effective, to cause resistance) in the patient’s disease (corresponding to AMP/ASCO/CAP Tier IA).

CVI score 6 (Clinical): The biomarker has not yet been approved by a regulatory agency for the patient’s disease, but the following evidence exists for patients with the same or a different disease:

(a) it predicted drug efficacy in at least one large cohort study (response), (corresponding to AMP/ASCO/CAP Tier IB).
(b) it is associated with drug inefficacy based on a retrospective study and/or cumulative evidence (resistance), (corresponding to AMP/ASCO/CAP Tier IB).
(c) the biomarker has been approved by FDA, EMA and/or other regulatory agency for a different cancer entity (corresponding to AMP/ASCO/CAP Tier IIC).

CVI score 5 (Clinical): The biomarker has not yet been approved by a regulatory agency for the patient’s disease. It has been observed to predict a specific effect of the drug (i.e., to be effective or to cause resistance) in small cohort studies or several case reports in patients with the same or a different disease (corresponding to AMP/ASCO/CAP Tiers IB, IID).

CVI score 4 (Clinical): The biomarker has not yet been approved by a regulatory agency for the patient’s disease. It has been observed to predict a specific effect of the drug (i.e., to be effective or to cause resistance) in single case reports in patients with the same or a different disease either with or without supporting preclinical evidence (corresponding to AMP/ASCO/CAP Tiers IID, III).

CVI score 3 (Preclinical): The biomarker has not yet been observed in patients to predict a specific effect of a drug. The biomarker has been observed in preclinical experiments, e.g., in cell lines or mouse models (corresponding to AMP/ASCO/CAP Tier IID).

CVI score 2 (Preclinical): The biomarker has not yet been observed/tested in patients or preclinical models to predict a specific effect of an investigational drug, but the predicted effect is based on a biological rationale due to specific data for a variant with a similar effect on protein function (no corresponding AMP/ASCO/CAP Tier).

CVI score 1 (Preclinical): The biomarker has not yet been observed/tested in patients or preclinical models to predict a specific effect of an investigational drug, but the predicted effect is based on a biological rationale due to specific data for a variant with a similar effect on protein function. Only used in combination with drug classes (no corresponding AMP/ASCO/CAP Tier).

### 2.4. Ethics

Institutional Review Board Statement and Informed Consent Statement: Tissue/tumor samples and/or patient data used in this study were provided by the University Cancer Centre Frankfurt (UCT). The study was approved by the institutional Review Boards of the UCT and the Ethical Committee at the University Hospital Frankfurt (project-number: SDO-1-2025).

## 3. Results

28 clinically diagnosed malignant melanoma of the skin of 17 women (61%) and 11 men (39%) at the age of 23 to 85 years (median: 63 years) were analyzed. 7/28 (25%) malignant melanomas were diagnosed at the age of ≤50 years and 21/28 (75%) at the age of ≥50 years. As summarized in Table 3, tumors originate from various cutaneous, mucosal, and metastatic sites, such as the anal canal, lymph nodes, spine, and extremities. The predominant diagnosis is malignant melanoma or its subtypes (e.g., nodular melanoma, amelanotic melanoma), with some entries specifying metastatic or recurrent lesions. Tumor cell content varies from 40% to 95%, reflecting differences in sample purity and tumor burden but sufficient for a complex molecular diagnostic.

### Molecular Landscape of Detected Class 3–5 Variants

**Figure 1:**
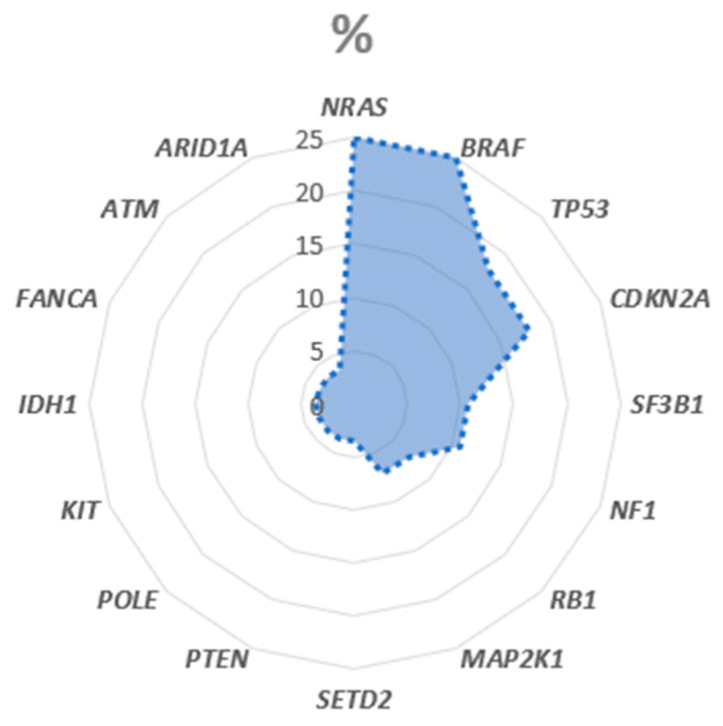
Pathogenic (P) and likely pathogenic (LP) variants (22/28). One tumor contains more than one P/LP variant.

79% (22/28) present a pathogenic or likely pathogenic variant with an allelic frequency of ≥5%. In total 42 somatic pathogenic or likely pathogenic variants with an allelic frequency of ≥5% could be detected, as visible in Figure 1. All variants were detected in tumor-only sequencing.

**Figure 2:**
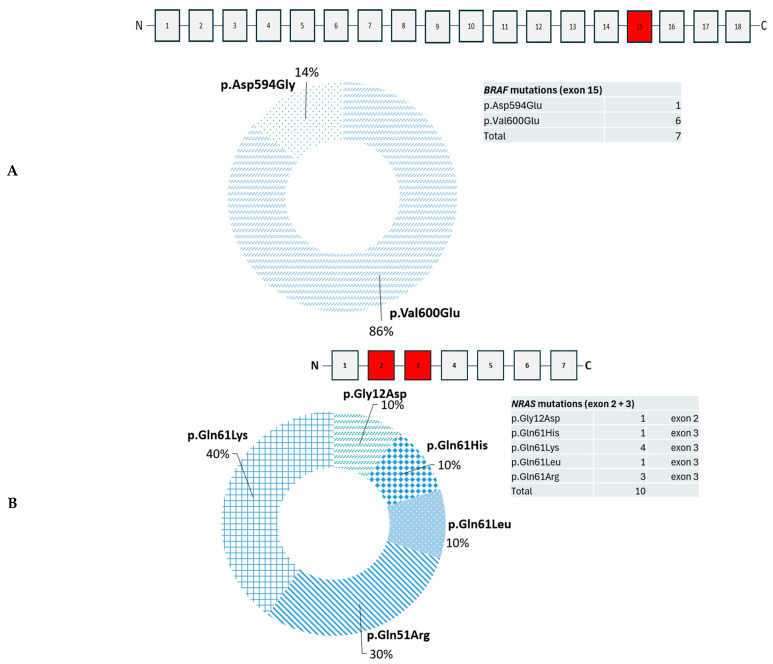
Summary of the frequency of pathogenic and likely pathogenic variants in *BRAF* (**A**) and *NRAS* (**B**) each 25% (7/28) altered. The affected exons are highlighted in red.

The most frequent molecular alteration in these melanomas were found in *NRAS* (25%) and *BRAF* (25%). The affected exons and respective variants are highlighted in Figure 2.

In addition, *TP53* (18%) and *CDKN2A* (18%) mutations are also found in the majority of cases followed by alterations in *SF3B* (11%), *NF1* (11%), *RB1* (7%) and *MAP2K1* (7%). *KIT* Exon 13 mutations (K642E) are found in a small subset of cases (4%).

*SETD2*, *PTEN*, *POLE*, *IDH1*, *FANCA*, *ATM* and *ARID1A* are altered in 4% of melanomas, respectively.

**Figure 3:**
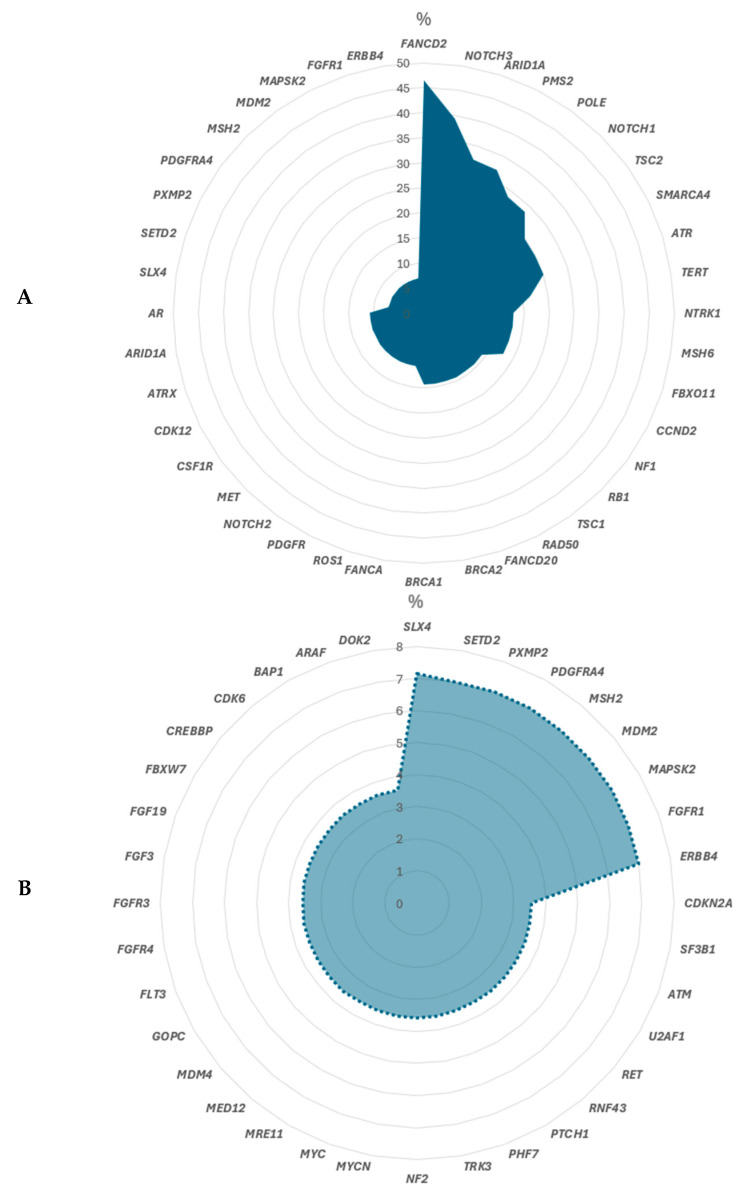
Variants of unknown significance (VUS). One tumor contains more than one variant. VUS with a higher frequency (**A**) and lower frequency (**B**) in malignant melanoma.

The most frequent molecular alteration of unknown significance was found in *FANDC2* (46%), *NOTCH3* (39%), *ARID1A* (32%), *PMS2* (32%), *POLE* (29%), *NOTCH1* (29%), *TSC2* (25%), *SMARCA4* (25%), *ATR* (25%) and *TERT* (21%), as shown in Figure 3A. Other variants of unknown significance which are less common are visible in Figure 3B.

For 36% of patients with a variant in *ATM*, *BRAF*, *FANCA*, *IDH1*, *KIT* or *PTEN* at least one well-powered clinical study with large patient numbers or approved by FDA and/or EMA for a different cancer entity was available (Table 4).

CVI Score 7 (approved therapies for the specific patient indication) is available for only 6 patients (18%) in the cohort and only for the *BRAF* biomarker (Table 5).

## 4. Discussion

Malignant Melanoma is an aggressive form of skin cancer with high metastatic potential and increasing incidence worldwide. Advances in molecular characterization have deepened our understanding of genetic alterations, especially mutations in *BRAF*, *NRAS*, and *KIT*, enabling targeted therapies. Immunotherapies such as checkpoint inhibitors (anti-CTLA-4, anti-PD-1/PD-L1) have significantly improved outcomes in advanced melanoma, though resistance and heterogeneous responses remain major challenges. This project analyzes a melanoma cohort using next-generation sequencing to identify relevant molecular patterns in tumor only. Pathogenic *BRAF* and *NRAS* mutations were most frequent; additionally, numerous other genes harbored variants. These findings underscore the genetic complexity of melanoma and its relevance for personalized therapy. Integrating genomic profiling with immunologic markers may improve patient stratification and guide combination strategies—such as targeted therapy followed by immunotherapy—for sustained disease control. As preliminary information, all variants were called from tumor-only sequencing data generated with the Oncomine™ Comprehensive Assay v3. No matched normal samples were sequenced, so variants could represent either somatic or germline alterations. Somatic variant reporting followed the ACMG/AMP guidelines for somatic NGS analysis, with prioritization of known oncogenic or likely pathogenic variants. As a result, germline variants cannot be definitively distinguished in this dataset, and dedicated germline testing would be required to identify inherited mutations, as also noted in the Section 4.

Testing for *BRAF*, *NRAS* and *KIT* mutations is recommended by ESMO, NCCN, S3 and Oncopedia guidelines in resectable or unresectable stage II and IV melanoma. *BRAF* V600 mutation status is the most critical marker for targeted therapies. PD-L1 testing is commonly performed to help guide immunotherapy decisions, though its predictive value can vary with respect to the response to immune checkpoint inhibitors (e.g., Pembrolizumab, Nivolumab). *NTRK* fusions are very rare (<1%) in melanoma but are important because targeted therapies are available. Testing is recommended by ESMO, NCCN, S3 and Oncopedia in selected cases, especially in advanced disease.

In our study, we also analyzed the mutation profiles of key genes such as *BRAF*, *NRAS*, *TP53* as well as *KIT* in malignant melanoma samples. These genes are critically involved in melanoma pathogenesis and have important implications for diagnosis, prognosis, and targeted therapy.

In this study, the most frequent molecular alteration in these malignant melanomas were found in *NRAS* (25%) and *BRAF* (25%). *KIT* Exon 13 mutations (K642E) are found in a small subset of cases (4%). According to the literature mutations in *BRAF* and *NRAS* occur more frequently as compared to other genetic alterations. In our malignant-melanoma cohort, *BRAF* and *NRAS* mutations each occurred in 25%, which is only slightly differently as reported in recent systemic review (*BRAF* ~38.5%, *NRAS* ~16.4% in a recent systematic review) [35]. These differences may reflect sample-selection bias—such as enrichment for specific clinical stages—as well as variation in melanoma subtype composition within our study population.

*BRAF* mutations, particularly the V600E variant, are among the most common genetic alterations in melanoma and have been successfully targeted with specific inhibitors, leading to improved patient outcomes [36]. These mutations lead to constitutive activation of the MAPK signaling pathway, promoting uncontrolled cellular proliferation. The introduction of selective BRAF inhibitors (e.g., vemurafenib, dabrafenib) revolutionized the therapeutic landscape for *BRAF*-mutant melanoma [37]. Clinical trials have demonstrated significant improvement in progression-free survival (PFS) and overall survival (OS) with these agents compared to chemotherapy. However, despite initial efficacy, resistance to BRAF inhibitors develops in a majority of patients within 6–12 months due to reactivation of MAPK signaling or alternative pathways [38]. Combination therapies with MEK inhibitors (e.g., trametinib, cobimetinib) have been shown to delay resistance, reduce toxicity, and further improve clinical outcomes [39].

Nevertheless, challenges remain as intratumoral heterogeneity and the evolution of resistant subclones highlight the complexity of melanoma biology.

The presence of *NRAS* mutations, which also occur frequently, influences tumor behavior and may impact response to certain therapies. The *NRAS* mutated tumors represent a distinct subset with poorer prognosis [40]. Unlike *BRAF* mutations, direct targeting of *NRAS*-mutant melanomas has proven difficult due to the intrinsic characteristics of RAS proteins. Currently, MEK inhibitors (e.g., binimetinib) provide limited clinical benefit, underscoring the need for alternative strategies. Emerging approaches, such as combination therapies targeting MEK and CDK4/6 or immune checkpoint inhibitors, show promise for *NRAS*-mutant melanomas. For instance, the use of immune checkpoint inhibitors like anti-PD-1 (nivolumab, pembrolizumab) and anti-CTLA-4 (ipilimumab) has demonstrated durable responses regardless of *NRAS* mutation status [41]. However, further research is needed to optimize treatment combinations and identify predictive biomarkers for therapeutic response.

In our study pathogenic/likely pathogenic mutations were present in *TP53* and *CDKN2A* in 18% of malignant melanomas and are according to the literature associated with increased tumor aggressiveness and poorer prognosis [42]. These alterations often reflect genomic instability and may serve as markers for disease progression. We detected *KIT* mutations (4%) which are predominantly observed in specific melanoma subtypes, such as acral and mucosal melanomas, and represent potential targets for kinase inhibitor therapies [43].

In addition to the well-characterized mutations in genes such as *BRAF*, *NRAS*, *TP53*, *CDKN2A*, and *KIT*, our study also highlights the significance of pathogenic/likely pathogenic alterations in *SF3B1*, *NF1*, *RB1*, and *MAP2K1* in malignant melanoma.

*SF3B1* mutations, although less common, have been increasingly recognized in melanoma and are thought to play a role in RNA splicing dysregulation, contributing to tumor progression. Their presence may also have potential as prognostic markers or therapeutic targets, although further research is needed to clarify their exact role. *NF1* acts as a tumor suppressor gene that negatively regulates RAS signaling pathways. Loss-of-function mutations in *NF1* are associated with a subset of melanomas, particularly those lacking *BRAF* or *NRAS* mutations. These alterations can lead to increased RAS pathway activity, promoting tumor growth and potentially influencing response to targeted therapies [44].

*RB1* is a critical regulator of cell cycle progression. Mutations or deletions in *RB1* can result in uncontrolled cellular proliferation, contributing to melanoma aggressiveness. The status of *RB1* may also impact the effectiveness of certain therapeutic agents, making it a gene of interest for future studies [45]. *MAP2K1* encodes MEK1, a key component of the MAPK signaling pathway. Mutations in *MAP2K1* can activate this pathway independently of upstream mutations, such as *BRAF* or *NRAS*, and may confer resistance to targeted therapies. Understanding these alterations can help in designing combination treatments or overcoming resistance mechanisms [46].

Overall, the inclusion of *SF3B1*, *NF1*, *RB1*, and *MAP2K1* in the mutational landscape of melanoma underscores the genetic complexity of this disease. These genes may serve as additional biomarkers for prognosis or as novel therapeutic targets, emphasizing the need for comprehensive genomic profiling in melanoma management.

In the context of malignant melanoma, some clearly defined pathogenic or likely pathogenic variants are found which are an essential tool for understanding tumor biology and guiding targeted therapies. However, the identification of variants of unknown significance (VUS) presents both challenges and opportunities. The most frequent molecular alteration of unknown significance was found in *FANDC2* (46%), *NOTCH3* (39%), *ARID1A* (32%), *PMS2* (32%), *POLE* (29%), *NOTCH1* (29%), *TSC2* (25%), *SMARCA4* (25%), *ATR* (25%) and *TERT* (21%). VUS are genetic alterations whose impact on protein function and disease progression is not yet clearly understood. As such, they represent a frontier in melanoma research, highlighting the need for ongoing functional studies and larger genomic databases to clarify their roles. Distinguishing somatic from germline VUS is essential for accurate risk assessment, familial counseling, and potential targeted therapy decisions. Matched-normal analyses and allele frequency context are critical. To detect a germline variant, the analysis would have to be performed on the blood of the respective patient. The testing takes place as part of a human genetics consultation. The presence of VUS complicates clinical decision-making, as it is difficult to determine whether these variants contribute to tumor development, influence treatment response, or affect prognosis. Nevertheless, these variants also offer a valuable resource for future research, as they may uncover novel pathogenic mechanisms or therapeutic targets once their significance is elucidated. Many VUS may impact signaling pathways relevant to melanoma (e.g., MAPK, PI3K–AKT) or cell cycle control, but require functional validation. Variants that are recurrent, predicted by multiple in silico tools to disrupt protein function, or present in high-frequency subclones should be prioritized for functional validation. Recommended approaches include pathway activation assays, proliferation and apoptosis studies, and drug sensitivity testing using CRISPR-edited cell models or patient-derived systems.

In vitro assays and model systems can help determine effects on proliferation, apoptosis, and drug sensitivity. According to Manganelli and colleagues, the mutation landscapes of melanomas should also be discussed in terms of UV-induced mutagenesis in connection with oncogenic signaling and immune interactions [9]. Environmental and genomic factors may co-modulate the relevance of VUS in MAPK and PI3K–AKT pathways and influence therapeutic responses to targeted inhibitors and immunotherapies. Future work should incorporate multi-omic integration with environmental exposure data and cross-cohort analyses to elevate VUS prioritization, enabling more precise risk assessment and personalized management. Collaborative consortia, variant curation expert panels, and shared functional genomics platforms can facilitate consensus interpretation and accelerate the classification of clinically meaningful variants. Clinical reports should clearly label variants as “Variants of Uncertain Significance” and avoid implying pathogenicity or directing changes in management based solely on the finding. Annotation flags can indicate uncertain functional impact, conflicting in silico predictions, low population frequency, or potential involvement in relevant signaling pathways. Follow-up recommendations should be provided, such as considering germline testing if indicated, re-reviewing the variant periodically, or enrolling the patient in research or functional studies. Reports should also note whether the variant has been submitted to shared databases like ClinVar or OMIM to facilitate ongoing data sharing and reclassification. In the molecular tumor board, these cases are discussed in detail in an interdisciplinary manner and further procedures are agreed upon jointly. The heterogeneity of melanoma suggests that personalized combination therapies will be essential to overcome resistance mechanisms. Novel agents targeting pathways such as ERK, PI3K/AKT, or immunometabolic pathways could complement current therapies [47].

### Study Limitation

A limitation of this study is the small cohort size and the limited availability of detailed clinical information for the melanoma patients included in the dataset. Apart from basic demographic and histopathological parameters—such as age, sex, tumor localization, and diagnosis—no comprehensive clinical follow-up data (e.g., disease stage, treatment history, response, or survival outcomes) were available. This restricts the ability to correlate molecular or pathological findings with prognostic or therapeutic parameters. The absence of extended clinical data is mainly due to the retrospective and multi-institutional nature of sample collection, where full clinical documentation was not consistently accessible. Matched-normal samples were not available, preventing definitive discrimination between somatic and germline variants. Technical limitations include potential artifacts from formalin-fixed, paraffin-embedded (FFPE) tissue and the known challenges of the Ion Torrent platform in accurately detecting homopolymer-associated indels. Additionally, samples were collected over several years (2017–2022), raising the possibility of batch effects or temporal heterogeneity that could influence variant detection and allele frequencies. These factors should be considered when interpreting variant prevalence, pathway involvement, and potential clinical implications.

## 5. Conclusions

The genetic landscape of malignant melanoma is highly diverse and complex. Malignant melanoma exhibits substantial molecular heterogeneity, with frequent pathogenic variants in *BRAF*, *NRAS*, *TP53*, *CDKN2A*, *KIT*, *SF3B1*, *NF1*, *RB1*, and *MAP2K1*, alongside numerous variants of unknown significance. These findings reinforce the importance of integrating genetic profiling with immunologic (PD-L1) and histopathological markers to guide personalized therapy. Moving forward, we plan to expand the cohort to include diverse histological subtypes and perform germline testing in correlation to clinical parameters to translate molecular insights into robust, individualized treatment strategies within the next 24 months.

## Figures and Tables

**Table 1:** Overview of molecular biomarkers and therapeutic approaches.

Marker	Frequency	Function	Therapeutic Implication
*BRAF* V600E	~40–60%	Activates MAPK/ERK pathway	BRAF ± MEK inhibitors (e.g., vemurafenib, dabrafenib)
*NRAS*	~15–20%	Activates MAPK and PI3K/AKT pathways	MEK inhibitors (off-label use)
*KIT*	<5% (mucosal/acral)	RTK mutations/amplifications	KIT inhibitors (e.g., imatinib, nilotinib)
*PTEN*	Loss in ~10–20%	Tumor suppressor, regulates PI3K/AKT	Associated with resistance to therapy
*TP53*	~15%	Tumor suppressor gene	Poor prognosis indicator
*CDKN2A*	Familial melanoma (20–40%), ~2% in melanoma cases without a clear family history	Cell cycle regulation	Risk prediction; limited therapy role
PD-L1	Variable, varying PD-L1 expression, with some studies reporting rates around 38–62%	Immune evasion via T-cell inhibition	Predictor for immunotherapy response (e.g., Pembrolizumab, Nivolumab)

**Table 2:** Oncomine™ Comprehensive DNA Assay v3 enables the detection of SNVs and CNVs.

Hotspot Genes (87), SNVs				Copy Number Variants (43), CNVs	
*AKT1 AKT2 AKT3* *ALK AR ARAF* *AXL BRAF BTK* *CBL CCND1 CDK4 CDK6 CHEK2 CSF1R CTNNB1 DDR2 EGFR ERBB2 ERBB3 ERBB4 ERCC2*	*ESR1 EZH2 FGFR1 FGFR2 FGFR3 FGFR4 FLT3 FOXL2 GATA2 GNA11 GNAQ GNAS H3F3A HIST1H3B HNF1A HRAS IDH1 IDH2 JAK1 JAK2 JAK3* *KDR*	*KIT KNSTRN KRAS MAGOH MAP2K1 MAP2K2 MAP2K4 MAPK1 MAX MDM4 MED12 MET MTOR MYC MYCN MYD88 NFE2L2 NRAS NTRK1 NTRK2 NTRK3 PDGFRA*	PDGFRB PIK3CA PIK3CB PPP2R1A PTPN11 RAC1 RAF1 RET RHEB RHOA ROS1 SF3B1 SMAD4 SMO SPOP SRC STAT3 TERT * TOP1 U2AF1 XPO1	*AKT1 AKT2 AKT3 ALK AR AXL BRAF CCND1 CCND2 CCND3 CCNE1 CDK2 CDK4 CDK6 EGFR ERBB2 ESR1 FGF19 FGF3 FGFR1 FGFR2 FGFR3*	*FGFR4 FLT3 IGF1R KIT KRAS MDM2 MDM4 MET MYC MYCL MYCN NTRK1 NTRK2 NTRK3 PDGFRA PDGFRB PIK3CA PIK3CB PPARG RICTOR TERT*

* The Oncomine™ Comprehensive Assay v3 includes coverage of hotspot mutations in the TERT promoter. TERT promoter variants were called using the same Ion Reporter workflow and thresholds applied to coding regions (minimum AF ≥ 5%, coverage ≥ 500x). Due to the high GC content and known sequencing challenges in promoter regions, all TERT promoter calls were manually reviewed in IGV to ensure accuracy and minimize false positives.

**Table 3:** Overview of demographic and histopathological parameters.

Sample ID	Age	Sex	Localization	Histopathological Diagnosis	TCC [%]
MM1	70	m	Right axillary lymph node	Metastatic malignant melanoma	80
MM2	85	f	Anal canal	Malignant melanoma	80
MM3	67	f	Anal canal	Malignant melanoma	90
MM5	53	f	Right axillary lymph node	Malignant melanoma	90
MM7	29	f	Left knee	Metastasis of a malignant melanoma	60
MM13	61	f	Possibly skeletal lesion of the thoracic spine	Nodular malignant melanoma	60
MM14	58	m	Pretibial, left	Nodular melanoma (DD: nodular basal cell carcinoma)	80
MM16	66	f	Upper lumbar spine	Malignant melanoma	70
MM18	38	f	n.s.	Nodular malignant melanoma	20
MM19	71	f	Left thigh, ventral	Nodular malignant melanoma	80
MM20	67	m	Dermal, cervical left	Nodular malignant melanoma	80
MM21	79	f	Left forearm	Melanoma metastasis	70
MM22	83	f	Left ankle	Malignant melanoma	80
MM23	81	f	Left inguinal lymph node (history of breast carcinoma)	Nodal metastasis of a malignant melanoma	95
MM24	46	m	Right cheek	Malignant melanoma	80
MM25	53	f	Hard palate	Malignant melanoma	95
MM26	42	m	Left knee	Malignant melanoma	80
MM27	68	m	Thoracic spine	Malignant melanoma	80
MM28	34	m	Left flank	Malignant melanoma	85
MM30	83	m	Left inferior nasal turbinate	Malignant melanoma	70
MM32	57	f	Supra-anal	Amelanotic epithelioid malignant melanoma	80
MM33	73	f	Anal	Recurrent anal melanoma	80
MM34	34	m	Lumbar spine	Nodular superficially spreading malignant melanoma	50
MM36	23	m	Right thoracic back	Malignant melanoma	60
MM37	61	f	Melanoma of the trunk	Malignant melanoma	70
MM38	64	f	n.s.	Malignant melanoma	90
MM39	53	f	Cervical portio	Malignant melanoma	60
MM40	82	m	Urinary bladder	Malignant melanoma	40

All histological diagnoses were confirmed by pathological review. n.s. = not specified; DD = differential diagnosis; m = male; f = female.

**Table 4:** MH based CVI Score.

CVI Score	Patient Cohort Malignant Melanoma (*n* = 28)
CVI = 7 (Clinically approved)	6
CVI = 6 (Treatable alterations)	4
CVI < 6 (No treatable alterations)	18

**Table 5:** Treatment options for *BRAF* positive malignant melanomas.

Gene Symbol	Variant Symbol	Impact	Treatment	CVI Score	AMP Score
*BRAF*	p.V600E	Effective	Binimetinib (On Label) and Encorafenib (On Label)	7	IA
*BRAF*	p.V600E	Effective	Cobimetinib (On Label) and Atezolizumab (Off Label)&Vemurafenib (On Label)	7	IA
*BRAF*	p.V600E	Effective	Cobimetinib (On Label) and Vemurafenib (On Label)	7	IA
*BRAF*	p.V600E	Effective	Dabrafenib (On Label)	7	IA
*BRAF*	p.V600E	Effective	Trametinib (On Label)	7	IA
*BRAF*	p.V600E	Effective	Trametinib (On Label) and Dabrafenib (On Label)	7	IA
*BRAF*	p.V600E	Effective	Vemurafenib (On Label)	7	IA

## Data Availability

The original contributions presented in this study are included in the article. Further inquiries can be directed to the corresponding author.
